# Unlocking the outcomes of *Syzygium cumini* concentrate marination on the physicochemical and sensorial properties, oxidative and microbial stability of beef

**DOI:** 10.1016/j.fochx.2026.104673

**Published:** 2026-10-23

**Authors:** Muhammad Asad Ali Khan, Raheel Suleman, Muhammad Muzamil, Noor-Ul-Huda, Tariq Ismail, Muhammad Aftab Zahoor, Huan Liu, Moneera O. Aljobair, Isam A. Mohamed Ahmed

**Affiliations:** a Department of Food Science and Technology, Faculty of Food Science and Nutrition, Bahauddin Zakariya University, Multan 60800, Pakistan; b Institute of Food Science and Technology, Chinese Academy of Agricultural Sciences, Beijing 100193, China; c National Institute of Food Science and Technology, University of Agriculture Faisalabad, Faisalabad 38000, Pakistan; d School of Food Engineering, Yantai Key Laboratory of Nanoscience and Technology for Prepared Food, Yantai Engineering Research Center of Green Food Processing and Quality Control, Bionanotechnology Institute, Ludong University, Yantai, China; e Department of Sports Health, College of Sports Sciences and Physical Activity, Princess Nourah Bint Abdulrahman University, Riyadh, Saudi Arabia; f Department of Food Science and Nutrition, College of Food and Agricultural Sciences, King Saud University, Riyadh, Saudi Arabia

**Keywords:** Beef, *Syzygium cumini*, Marination, Microbial stability, Lipid oxidation

## Abstract

This study investigated the effects of *Syzygium cumini* concentrate marination on the physicochemical, sensory, oxidative, and microbial stability of beef, comparing BMS_1_-BMS_3_ (marination for 1–3 h) with the unmarinated control (B_0_). Chromatic values slightly decreased with increase in marination time. Marination improved textural properties significantly (*p* *<* *0.05*) by reducing hardness (58.8–34.1), gumminess (21.6–17.1), chewiness (15.4–14) and cooking loss (31.4–21.4) of beef samples from B_0_-BMS_3_. By the end of storage period (day-9), pH values reduced 5.7–5.5 among the treated samples for BMS_1_-BMS_3_. Simultaneously, oxidative stability improved, with reduction in TBARS (1.9–1.4 mg MDA/kg) in BMS_1_-BMS_3_ than control (2.6 mg MDA/kg). Marinated samples showed lower total plate counts, *E. coli* and *S. aureus* (4.4, 1.5 and 2.1) against control (5.4, 2.3 and 3.3) Log_10_ CFU/g. SEM and FTIR reported that marination improved beef surface morphology and compositional characteristics. Additionally, BMS_2_ showed highest sensory acceptance, whereas BMS_3_ depicted strongest preservation effects. PCA and correlation analysis revealed associations among the physicochemical, sensory, oxidative, and microbiological parameters, consistent with improved quality preservation in marinated beef during refrigerated storage.

## Highlights

• *Syzygium cumini* marinade improved beef quality and shelf life.
• Marination reduced lipid oxidation and microbial growth during storage.
• Enhanced tenderness and lowered cooking loss in treated beef.
• PCA and correlation revealed strong quality parameter relationships.
• SEM and FTIR revealed improved surface and compositional morphology.

## Graphical abstract

**Figure:**
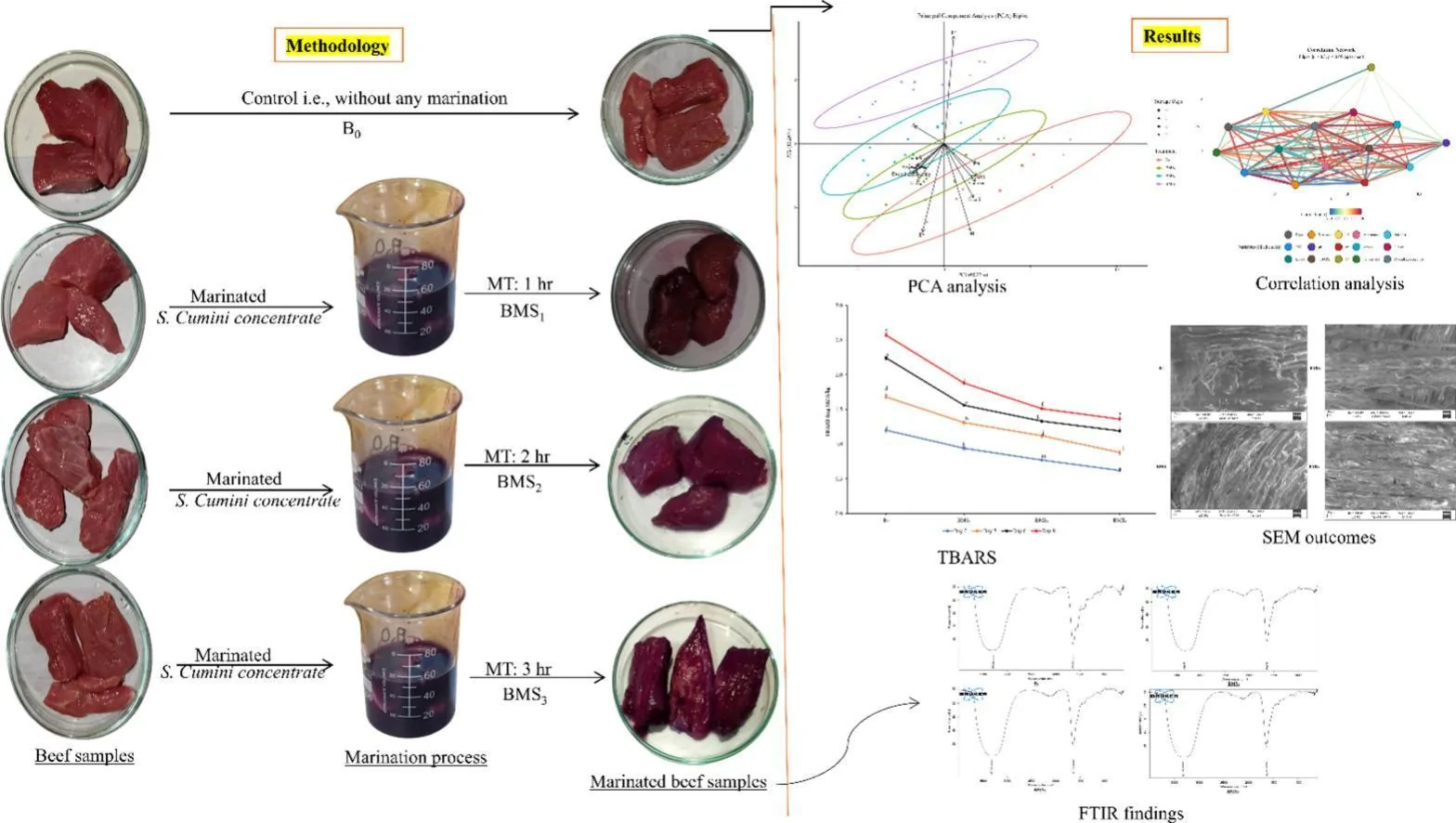

## 1 Introduction

Jamun *(Syzygium cumini)* is a tropical fruit belonging to the family (*Myrtaceae*) and genus (*Syzygium*), recognized with different names (Indian blackberry, jambolan, java plum, black plum and jambul) (Jans et al., 2026; Kumar et al., 2022). Major growers of this fruit are Pakistan, India, China, Sri Lanka, Bangladesh, USA, Algeria, Eastern Australia and Israel. Moreover, China is the largest producer of this fruit and estimated total production is 13.5 million tons per year (Kishore, 2019; Madani et al., 2021). It is enriched with macronutrients (carbohydrate, protein, fiber, fat) and micronutrients (potassium, magnesium, phosphorus, sodium, calcium, iron, zinc) (Singh et al., 2022). It also contains free amino acids (alanine, asparagine, tyrosine, glutamine, cysteine), vitamins (ascorbic acid, niacin, riboflavin, thiamine) and organic acids (betulic acid, oxalic acid, gallic acid, tannic acid, malic acid) (Shah et al., 2021). It is an excellent source of bioactive compounds (ellagic acid, terpenoids, alkaloids, tannins, anthocyanins, quercetin, kaempferol, cyanidin, malvidin, delphinidin) that exhibit antioxidant and antimicrobial properties (Ayenampudi et al., 2023). It provides health benefits such as hepatoprotective activity, antiallergic, anti-inflammatory, improved central nervous system and human metabolism (Rizvi et al., 2022).

However, this fruit is still underutilized due to its purple stain creating characteristics on the tongue, acidic nature and astringent flavor; although, it is enriched with the notable nutritional profile. This fruit has juicy and delicate texture, and its highly perishable nature causes postharvest losses (El-Safy et al., 2023). Therefore, it has short shelf life of 1–2 days at the ambient storage and remains available only for specific season (Kumar et al., 2022). To combat such issues, utilization of this fruit into various food applications, is the only way to add value and make use of this fruit economically in an effective way (El-Safy et al., 2023). Beef is essential component of human diet and enriched with macronutrients, saturated and unsaturated fatty acids, high quality protein and water. It contains micronutrients including vitamins (i.e., cobalamin, pyridoxin, pantothenic acid, niacin, riboflavin, thiamine, vitamin D) and minerals (i.e., selenium, zinc, iron) (Soltana et al., 2024). Meat and meat products are highly perishable and vulnerable to spoilage due to its high nutrient content, water activity and pH (Farokhzad et al., 2023). The raw meat is highly susceptible to the oxidative and microbial spoilage, results in reduction of nutritional value and loss of flavor, texture and color pigments (Bukvicki et al., 2023). Currently, meat industries are facing main issue of increasing general acceptability and shelf-life of meat by controlling microbial growth and retarding lipid oxidation (Isvand et al., 2024).

To resolve these issues, chemical additives are added in meat and meat products during processing (i.e., butylated hydroxytoluene and butylated hydroxyanisole). But, utilization of synthetic additives is still questionable, due to their associated risks of increasing toxicity in meat and meat-based products (Jaworska et al., 2021). The excessive utilization of synthetic additives have increased concerns regarding potential adverse health effects (Aldabayan, 2025). Therefore, researchers are increasingly exploring natural preservation approaches that may help maintain food quality and stability during storage (Shah Hosseini, 2023). Among previous studies, Talukder et al. (2020) prepared a freshness indicator through incorporation of *Syzygium cumini* skin extract, for investigating variations in quality of chicken patties at refrigerated storage. Barbosa et al. (2022) developed *Syzygium cumini* incorporated packaging film to enhance oxidative quality of lamb burgers. Another study Fatima et al. (2019) prevented from deterioration and enhanced quality characteristics of *Litopenaeus vannamei* (shrimp) through *Syzygium cumini* leaf-extract at cold storage. Mentioned findings revealed *Syzygium cumini* potential as a natural available source of bioactive compounds to enhance perishable food products quality and stability.

Beef is highly susceptible to microbial proliferation and oxidative deterioration, which can adversely affect its sensory acceptability and overall quality during storage. Some synthetic antioxidants and preservatives are usually utilized to control deteriorative reactions, but raising trends regarding synthetic additives adverse health effects stimulating consumer and researchers' interest in natural preservation approaches. Furthermore, there is no study available in literature which investigated *Syzygium cumini* as a marinade for exploring the preservation of meat and meat-based products. Therefore, the present research was planned to explore the potential of *Syzygium cumini* concentrate as natural plant-based marinade to improve the technological characteristics, antimicrobial and antioxidative properties of beef at refrigerated storage.

## 2 Material and methods

### 2.1 Procurement of chemicals, reagents and raw material

Fresh boneless beef of the thigh muscles were obtained from three healthy male Sahiwal breed cattle, were acquired from the Metro Mall, Multan, Punjab, Pakistan. The meat was received at 24 h postmortem and taken to the Food Analysis Lab, Department of Food Science and Technology, Bahauddin Zakariya University, Multan, Pakistan, at refrigerated conditions. *Syzygium cumini* fruits were purchased from the local fruits and vegetables market of Multan, Pakistan. All the chemicals and reagents used in this research experiment were of analytical grade and were purchased from the authorized suppliers. Chemicals and reagents i.e., hydrochloric acid, trichloroacetic acid, Thiobarbituric acid reagent, saline water, nutrient agar, eosin methylene blue agar, and mannitol salt agar were purchased from the university authorized distributors of Sigma-Aldrich Corporation (St. Louis, MO, USA) and Merck (Darmstadt, Germany).

### 2.2 Preparation of *Syzygium cumini* concentrate

Fresh *Syzygium cumini* (Desi Jamun) were collected from Multan fruits market and washed thoroughly under running tap water to detach surface impurities. The seeds were manually discarded and pulp was allowed to be homogenized by utilizing a laboratory blender (Model = J-W176P, Panasonic Corporation, Osaka, Japan) to obtain a pure juice (Malik et al., 2023). Afterwards, *Syzygium cumini* fruit juice was concentrated in a laboratory rotary evaporator (Model = RE100-S LED Digital Rotary Evaporator, DLAB Scientific Co., Ltd., Beijing, China) at temperature of 45–50 °C to achieve the desired consistency (30% remaining concentrate obtained). The end-point concentration of concentrate consistency was adopted as a practical processing criterion to develop sufficiently concentrated, functionally flowable concentrate to use it as a natural marinade for meat. Then the prepared *Syzygium cumini* concentrates were stored in the sterilized glass containers at 4 °C for further utilization (Saad et al., 2021; Saleem et al., 2025).

### 2.3 Characterization of *Syzygium cumini* concentrate

#### 2.3.1 pH, titratable acidity and total soluble solids determination

pH of *Syzygium cumini* concentrate was investigated with the help of pH meter (Sentron model 1001, Leek, The Netherlands) by directly dipping electrodes into the concentrate. Titratable acidity of concentrate was determined by titrating it with 0.1 N NaOH, by inserting few drops of phenolphthalein as an indicator. Results were calculated by noting volumetric readings when light pink color appeared as performed by (Jan et al., 2025). Total soluble solids were measured by employing refractometer (MRC K7135) and presented as a degree Brix (^0^B) (Jan et al., 2025).

#### 2.3.2 Antioxidant activity, total phenolic content and total flavonoids content estimation

The antioxidant activity was investigated by using DPPH radical scavenging activity. Precisely, 100 μL of sufficiently diluted *Syzygium cumini* concentrate was dissolved with 290 μL of 0.1 mM DPPH solution. This mixture was placed at dark place for incubation for 1 h after proper mixing. UV–Visible spectrophotometer (Specord 200 Plus, Analytik Jena, Germany) was utilized to measure the absorbance at 517 nm and results were measured as performed by (Jan et al., 2025) with slight modifications. To measure total phenolic content, 20 μL of diluted concentrate was dissolved with 1.58 mL distilled water and 100 μL of Folin-Ciocalteu reagent. After that, 300 μL of 20% Na2CO3 solution was inserted and mixture was placed for incubation at 20 °C for 2 h. Absorbance was measured at 765 nm and gallic acid was assimilated as calibration standard and results were presented as mg GAE/mL (Jan et al., 2025). Furthermore, to estimate total phenolic content 1 mL of diluted concentrate was dissolved with 5% NaNO2 in 300 μL, after that 600 μL of 10% AlCl3 was added. Then, 4.3% prepared NaOH was added in 2 mL volume and absorbance was calculated at 510 nm. Quercetin was assimilated as calibration standard and results were depicted as QE/mL as described by (Jan et al., 2025).

### 2.4 Beef samples preparation and implication of *Syzygium cumini* concentrate on beef

Fresh beef samples were acquired within 2–3 h before experimentation, trimmed to remove connective tissues, visible fat and placed in refrigerator i.e., 4 °C for subsequent analysis. Meat was cut into uniform pieces of approximately 20 g to maintain consistency across the treatments. For the preparation of each treatment, three independent biological replicates were made (*n* = 3) by collecting samples through three animals with one independent sample from thigh muscle of each animal meat. Then, beef samples were divided into 4 treatment groups including B_0_ (control) = beef without any marination, BMS_1_ = Beef marinated with *Syzygium cumini* concentrate for 1 h, BMS_2_ = Beef marinated with *Syzygium cumini* concentrate for 2 h, BMS_3_ = Beef marinated with *Syzygium cumini* concentrate for 3 h. Marination process was executed by directly submerging the beef samples in the concentrate at a constant meat-to-concentrate ratio i.e., 1:2 *w*/*v*, at temperature 4 °C. After execution of specific marination times, samples were removed from the *Syzygium cumini* concentrate and allowed to drain for a standardized period i.e., 5 min, and then packaged in polyethylene (PE) zipper lock bags. Moreover, air was manually evacuated from the PE bags before sealing and then packaged beef samples were placed at storage conditions (4 °C temperature, 75% relative humidity) for further beef storage stability investigations. Each beef sample was subjected to microbial, physicochemical, and sensorial analysis at 0–9 days of storage intervals (Çelik et al., 2025).

### 2.5 Colorimetric properties determination of beef

The chromatic profile of beef samples marinated with *Syzygium cumini* concentrate were estimated by utilizing an instrument colorimeter (Model number = Konica, Minolta CR 400, Osaka, Japan). This equipment is equipped with diffuse/O mode, 8 mm illumination-based aperture, measurement aperture, 2^0^ observer angle and illuminant D65 as delineated by Dilek et al. (2026). Before readings estimation, instrument was calibrated with the assistance of black and white plates. After that, beef sample placed under the lens of colorimeter and readings were measured for lightness (*L*^*⁎*^), redness (*a*^*⁎*^) and yellowness (*b*^*⁎*^) (Dilek et al., 2026).

### 2.6 Physicochemical characterization of beef

#### 2.6.1 Texture profile analysis

For texture analysis, 15 mm diameter measuring raw meat pieces were cut parallel according to the longitudinal orientation of meat muscles fiber and texture profile analysis were determined at room temperature (20 °C), by employing texture analyzer instrument (Texture Technologies Corp., Hamilton, MA, United States of America) (Saeed et al., 2026). For this purpose, 2-cycles compression test (i.e., 50%) was adopted by allowing the cylindrical flat probe (i.e., diameter = 100 mm) to press the sample at descending speed of 1.5 mm/s. Textural parameters including hardness, cohesiveness, springiness and gumminess were noted as detailed by (Lin et al., 2026).

#### 2.6.2 Cooking loss

Marinated beef samples were cooked at a temperature of 85–90 °C, until the core temperature of meat samples reached to 70 °C. The cooking loss of beef samples were investigated by the following formula, as presented by (Suleman, Choudhary, Waseem, et al., 2025).

(1) $$\text{Cooking loss}\;\left(\%\right)=\frac{\mathrm{Wa}-\mathrm{Wb}}{\mathrm{Wa}}\times 100$$

Wa = Weight of marinated beef sample prior to cooking, Wb = Weight of marinated beef sample after cooking.

#### 2.6.3 Water holding capacity

Water holding capacity of marinated beef samples were assessed by placing 5 g beef sample in between the two filter papers. After this, compression machine (Moel = YYW-2, Nanjing Soil Instrument, Nanjing, China) was employed to press the beef sample with force i.e., 343 N for 5 min. Water holding capacity was estimated by calculating the difference between weight of beef sample before compression and weight of beef sample after compression and results were presented with % unit as expressed by (Ijaz et al., 2022).

(2) $$\mathrm{WHC}\;\left(\%\right)=\frac{\text{Weight after compression}}{\text{Intial weight}}\times 100$$

### 2.7 pH estimation of beef

pH of marinated beef samples were determined by inserting the immersion electrode of digital pH meter (Model = Sentron model 1001, Leek, The Netherlands) at 1.27 cm depth, directly into meat surface as described by (Carrillo-Lopez et al., 2022).

### 2.8 TBARS determination of beef samples

To measure the Thiobarbituric acid reactive substances (TBARS) values, 2 g of minced marinated beef samples were integrated with 18 mL of 10% perchloric acid and this mixture (i.e., 3.75 mg/mL) was added to the solution containing 0.2 mL of butylated hydroxyanisole +98% of ethanol. Then, this mixture was allowed to be homogenized (D1000-E, Benchmark) at 2775 ×*g* for 1 min, afterwards centrifuged (Model = Combi-514R, Hanil Science, Korea) at the conditions (1360 ×*g*, 4 °C, 10 min). Following this, supernatant layer was separated with filter paper (Whatman No. 1), and mixed with 2 mL of 20 mM TBA and placed for incubation in dark room for 16–17 h. Thereafter, spectrophotometer (Optizen TM POP, KLAB, Daejeon, Korea) was employed to measure the mixture at 531 nm by running a blank sample in parallel, and results were depicted as mg malondialdehyde (MDA)/kg as described by (Kim et al., 2022) with slight modifications.

(3) $$\text{TBARS}\;\left(\mathrm{mg}\;\mathrm{MDA}/\mathrm{kg}\right)=\frac{50\times \left(A-B\right)}{m}$$

A = absorbance of test solution, B = absorbance of blank reagent, m = mass of test portion, 50 = a valid factor if the volumetric flask volume is 25 mL and cuvette width is 10 mm.

### 2.9 Total plate counts, *Escherichia coli* (*E. coli*) and *Staphylococcus aureus* (*S. aureus*) determination of beef samples

For this purpose, 1 g marinated beef sample was homogenized in 10 mL distilled water with the assistance of stomacher (400 Circulator, Stomacher®). After, 1 mL of test solution was diluted with saline water in range up to 10^−6^. Following this, eosin methylene blue agar, mannitol salt agar and nutrient agar were allowed to be prepared for investigation of *Escherichia coli* (*E. coli*), *Staphylococcus aureus* (*S. aureus*) and total plate counts in beef. 1 mL of each serially diluted test solution was spread on the media containing petri plates via the assistance of glass spreaders. Afterwards, plates were placed for incubation at controlled temperature i.e., 37 °C for 24 h. Lastly, colonies were counted with the assistance of colony counter (Model = CC-J2, Infitek Ltd., Shandong city, China) and results showed as Log_10_ colony-forming units (CFU)/g as experimented by (Suleman, Choudhary, Garcia-Galicia, et al., 2025).

### 2.10 Sensory evaluation of beef samples

Marinated beef samples were cooked and sensory ratings were determined through nine-point hedonic scale (1 = strongly disliked and 9 = strongly liked) (Muzamil et al., 2024). 50 semi-trained panelists (25 female and 25 males; age 24–40) were hired from Faculty of Food Science and Nutrition at Bahauddin Zakariya University, Pakistan. All the panel members were master and doctoral degree researchers and rated each sample by giving one score among nine to each sensory parameter and individual scores were presented as mean ± standard deviation for each sample and storage day. Panel members were instructed about the rating criteria, evaluation procedure and sensory attributes before analysis. Coded samples were randomly presented to panelists, and one cooked beef piece of each treatment was presented to every panel member for investigations. Additionally, informed consent was taken from panel members and ethical approval was acquired from the ethical committee of Faculty of Food Science and Nutrition, BZU, Multan. Furthermore, noise free environment, sufficient lights, and drinking water were managed in the sensory evaluation place. Appearance, aroma, tenderness, juiciness, flavor and overall acceptability were investigated as analyzed by (Muzamil et al., 2025).

### 2.11 Principal component analysis and correlation analysis

Principal component analysis (PCA) and correlation analysis (CA)-based approaches were utilized to investigate the differences and linkages among the microbial, sensorial, physicochemical, and oxidative characteristics of beef samples marinated with *Syzygium cumini* concentrate in different time intervals. These analyses were conducted through R-programming by employing R software (4.5.2 version, 2025-10-31 UCRT, R Foundation for Stat. Computing) and RStudio software (2026.01.0 + 392, Posit Soft., PBC) as presented by (Javeria et al., 2026; Zheng et al., 2025). These approaches were used to visualize multivariate patterns and associations among the estimated variables and were not discussed as independent validation of samples influence.

### 2.12 Fourier transform infrared (FTIR) investigations

Meat were freeze-dried and converted into homogenous powder form. FTIR spectrophotometer (Model = BT 27, Bruker Corporation, City Massachusetts, USA) was used for experimentation and sample was directly placed on ATR crystal of equipment to investigate spectra. This spectrum includes wavenumber range 4000–400 cm^−1^ at room temperature through enabling 32 scans by adjusting resolution to 4 cm^−1^ as experimented by (Lestari et al., 2022).

### 2.13 Scanning electron microscopy (SEM) analysis

To investigate microstructures of marinated beef samples, scanning electron microscope (Model = Cube II SEM, Emcraft, South Korea) was adopted and analysis was carried out at Research Center, Lahore College Women University (LCWU), Lahore, Pakistan. Marinated meat samples were freeze dried and coated with a layer of gold and outcomes were generated at an accelerating voltage of 15 kV as experimented by (Wei et al., 2020).

### 2.14 Statistical analysis

All the analysis was investigated by using three independent biological replicates (*n* = 3) and outcomes were presented as mean ± standard deviation. Two-way ANOVA (analysis of variance) was adopted with factorial design to analyze treatment × storage days interactions through Statistix 8.1 (Analytical Software Co., St. Paul, Thalassa, United State of America) at *p* *<* *0.05*. Those parameters where only treatment effects was investigated, were evaluated through complete randomized design (CRD), one-way ANOVA via the Statistix 8.1 software. Small superscript alphabetical lettering represents the significance of data among the treatments and storage days in tables and figures. Moreover, R-software (4.5.2 version, 2025-10-31 UCRT, R Foundation for Stat. Computing) and RStudio (2026.01.0 + 392, Posit Soft., PBC) were assimilated to depict correlation analysis and principal component analysis.

## 3 Result and discussion

### 3.1 Physicochemical and functional properties of *Syzygium cumini* concentrate

The physicochemical properties such as pH, titratable acidity and total soluble solids of *Syzygium cumini* concentrate were reported 3.4, 1.1% and 31.2 Brix (Table 1). The acidic nature of concentrate was found consistent with the acidic nature of *Syzygium cumini*, as Ghosh et al. (2019) reported pH, and total soluble solids of *Syzygium cumini* juice, i.e., 3.6 and 18 Brix. Jan et al. (2025) reported pH for fresh *Syzygium cumini* juice i.e., 0.32%. The increased acidity was estimated in concentrate may be linked with concentration of naturally occurring organic acids because of water removal during rotary evaporation. In the same way, the higher total soluble solids may also be associated with the water removal during evaporation process at rotary evaporator, while the minerals, organic acids, sugar and other constituents retained and become more concentrated (Kong et al., 2025). Moreover, the functional attributes reported notable higher total phenolic content and total flavonoids concentrate, i.e., 48.4 mg GAE/mL and 28.3 mg QE/mL in *Syzygium cumini* concentrate (Table 1). In addition to this, higher DPPH scavenging activity was reported for *Syzygium cumini* concentrate, i.e., 77.1%. The higher phenolic and flavonoid content were found consistent with the high phytochemical richness of *Syzygium cumini*. The content of phenolic and flavonoids could be mostly depended on the fruit part, solvent selection, extraction procedure, maturity and cultivar type. For instance, fresh jamun juice contained 14.4 mg GAE/mL phenolic compounds and 8.3 mg QE/mL total flavonoids content, according to previous study (Jan et al., 2025). The high compound observed may be linked with the retention of phenolic and concentration of juice during the rotary evaporation process. Furthermore, processing conditions can also influence the composition and retention of phenolic compounds in *Syzygium cumini* juice (Sarbatly et al., 2023). Jan et al. (2025) reported DPPH scavenging activity of *Syzygium cumini* juice 53.4%. The higher DPPH scavenging activity estimated in concentrate may be associated with the synergistic contribution of flavonoids and phenolic compounds of *Syzygium cumini*. Further, the concentration of compounds through rotary evaporation process further enhanced the antioxidant capacity of this fruit juice concentrate (Qamar et al., 2022). As observed in this study, the higher acidity and antioxidant activity is prominently relevant to its application as a marinade because its higher antioxidant and other bioactive constituents contributes role in controlling oxidative stress during meat storage (Latoch et al., 2023).

**Table 1:** Physicochemical and functional composition of *Syzygium cumini* concentrate.

Parameters	Results
pH	3.40 ± 0.03^e^
Titratable acidity (%)	1.13 ± 0.06^f^
Total soluble solids (Brix)	31.20 ± 0.85^d^
DPPH scavenging activity (%)	77.12 ± 1.22^a^
Total phenolic content (mg GAE/mL)	48.40 ± 2.11^b^
Total flavonoids content (mg QE/mL)	28.30 ± 1.34^c^

Note: Results are depicted as mean ± standard deviations (SD). The superscript small letters in this table on the average values demonstrated significance of data among the results of different parameters investigated for *Syzygium cumini* concentrate.

### 3.2 Effect of *Syzygium cumini* concentrate marination on colorimetric properties of beef

The colorimetric results of beef marinated with *Syzygium cumini* concentrate in distinct time intervals reported significant decline (*p* *<* *0.05*) in *L*^*⁎*^, *a*^*⁎*^ values and increment in *b*^*⁎*^ values from 52.2 to 40.7, 8.4–6.9 and 13.6–16.4 for BMS_1_-BMS_3_ (i.e., beef marinated for 1 h – beef marinated for 3 h) at storage days of 0–9, respectively (Table 2). However, the control B_0_ (i.e., beef without marination) reported significant changes (*p* *<* *0.05*) in *L*^*⁎*^, *a*^*⁎*^ and *b*^*⁎*^ values i.e., 54–43.8, 7.6–4.1, and 12.1–15.9 at 0–9 storage days, respectively. The beef treatment BMS_2_ (i.e., marinated for 2 h) delineated best colorimetric results for *L*^*⁎*^, *a*^*⁎*^ and *b*^*⁎*^ values i.e., 52.1–41.7, 10.3–6.2 and 14.4–16.3 as compared to other all marinated beef samples. The results briefly elaborated that the beef samples showed significant decrease in *L*^*⁎*^ values and increase in *a*^*⁎*^, *b*^*⁎*^ values, with the increment in marination time for 1–3 h, over the storage of 0–9 days, respectively. Overall, marinated beef samples showed lower *L*^*⁎*^ values, but higher *a*^*⁎*^ and *b*^*⁎*^ values as contrasted to the beef without marination at final days of storage. Among previous researches, Dai et al. (2026) reported beef marinated with Chinese liquor demonstrated significant higher *L*^*⁎*^, *a*^*⁎*^ and *b*^*⁎*^ values from 41.4 to 35.9, 14–16.2 and 11–10.8 as contrasted to control (beef without any marination) i.e., 37.4–30.8, 22.2–13.3 and 12.7–7.6 at storage intervals of 0–9, respectively. In another research rooster meat was marinated with strawberry-based marinated solution which showed significant higher values of *L*^*⁎*^, *a*^*⁎*^ and *b*^*⁎*^ i.e., 10.4, 8.7 and 12.7 as differentiated to rooster without any marination i.e., 9.9, 6.8 and 10.2 as tabulated by (Yerli̇kaya et al., 2026). Another research investigated pork meat which was marinated with the sour cherry fruit-based marinade exhibited prominent higher chromatic values of *L*^*⁎*^, *a*^*⁎*^ and *b*^*⁎*^ i.e., 45–55, 22–10 and 1–8 as compared to control i.e., 48–43, 7–4 and 2–6 at 0–12 days of storage (Nour, 2022). The lower *L*^*⁎*^ values of marinated beef samples in relative to control could be associated with the marination of *Syzygium cumini* concentrate. It may link with the penetration of phenolic pigments (anthocyanins) from *Syzygium cumini* concentrate into meat-matrix and reduced light reflectance and enhance darker surface appearance. Additionally during marination, protein denaturation and higher water retention are also responsible for declining further light scattering on surface (Çelik et al., 2025). The reduction in *a*^*⁎*^ values over the storage period may be linked with the progressive myoglobin and pigment oxidation. However, the higher *a*^*⁎*^ values estimated in marinated beef samples may be due to purple-red pigments of *Syzygium cumini* concentrate, imparting color on the meat surface. Additionally, it may also be linked with antioxidant compounds especially anthocyanins that helps in stabilizing oxymyoglobin (Nour, 2022). The higher *b*^*⁎*^ values may be connected to the non-enzymatic browning reactions and interactions between muscle protein and phenolics over storage. This phenomena can attribute to shift suggesting higher yellowness readings (Han et al., 2024; Zhao et al., 2024). Additionally, the variations observed in our findings as compared to previous studies, could be depend on composition of marinade, methods of marination, meat type, processing and storage conditions which can impact on the outcomes of observed quality attributes (Suleman, Choudhary, Garcia-Galicia, et al., 2025).

**Table 2:** Effect of *Syzygium cumini* concentrate marination on the chromatic profile of beef at 0–9 days of storage.

		Days		
	0th	3rd	6th	9th
*L*^*⁎*^ values				
B_0_	54.04 ± 4.26^a^	50.21 ± 1.00^bc^	46.11 ± 2.02^ef^	43.78 ± 0.59^fgh^
BMS_1_	52.22 ± 0.18^ab^	50.14 ± 0.01^bc^	47.14 ± 0.99^de^	43.30 ± 1.15^gh^
BMS_2_	52.12 ± 1.69^ab^	49.32 ± 1.01^cd^	45.02 ± 1.01^efg^	41.96 ± 0.36^hi^
BMS_3_	51.04 ± 0.84^bc^	47.17 ± 2.01^de^	43.85 ± 0.64^fgh^	40.72 ± 0.61^i^
*a*^*⁎*^ values				
B_0_	7.62 ± 0.76b^cde^	6.21 ± 1.00^efg^	5.51 ± 1.00^fgh^	4.11 ± 1.00^h^
BMS_1_	8.36 ± 0.56^bc^	7.24 ± 1.01^bcde^	6.77 ± 0.06^def^	5.02 ± 1.00^gh^
BMS_2_	10.34 ± 1.44^a^	8.00 ± 0.59^bcd^	7.31 ± 1.00^bcde^	6.22 ± 0.99^efg^
BMS_3_	10.75 ± 0.84^a^	8.66 ± 1.00^b^	7.66 ± 1.02^bcde^	6.94 ± 0.03^cdef^
*b*^*⁎*^ values				
B_0_	12.13 ± 0.95^e^	14.04 ± 0.01^cd^	15.08 ± 0.02^abc^	15.90 ± 1.54^ab^
BMS_1_	13.55 ± 1.01^de^	14.12 ± 0.03^cd^	14.98 ± 1.00^abcd^	15.99 ± 1.01^a^
BMS_2_	14.44 ± 1.04^bcd^	15.17 ± 0.34^abc^	15.92 ± 0.05^a^	16.33 ± 2.01^a^
BMS_3_	15.32 ± 0.01^abc^	15.97 ± 1.01^a^	16.23 ± 0.02^a^	16.37 ± 0.02^a^

Note: Results are depicted as mean ± standard deviations (SD). The superscript lettering on the values demonstrated significant results among the means on storage in rows and treatments in column. B_0_ = Beef without marination. BMS_1_ = Beef marinated with *Syzygium cumini* concentrate for 1 h, BMS_2_ = Beef marinated with *Syzygium cumini* concentrate for 2 h, BMS_3_ = Beef marinated with *Syzygium cumini* concentrate for 3 h.

### 3.3 Effect of *Syzygium cumini* concentrate marination on texture profile of beef

The texture profile analysis reported significant decrease (*p* *<* *0.05*) in hardness, gumminess and chewiness for BMS_1_-BMS_3_ from 46.4 to 34.2 N, 20.9–17.1 N, and 15.7–14 N in comparison to control, i.e., 52.8, 21.7 and 15.4 N (Fig. 1). Moreover, marinated beef showed significant increase (*p* *<* *0.05*) in cohesiveness and springiness i.e., 0.45–0.5 and 0.75–0.82 for BMS_1_-BMS_3_ against control, i.e., 0.41 and 0.71. In addition, cooking loss showed significant decline for BMS_1_-BMS_3_ from 27.9 to 21.4% as compared to control (31.4%). Additionally, marinated beef samples also showed notable increment in water holding capacity from 62.7 to 70.9% for BMS_1_-BMS_3_, as contrasted to control i.e., 58.26%. Overall, beef samples marinated with *Syzygium cumini* concentrate demonstrated best results for hardness, cohesiveness, springiness, chewiness, cooking loss and water holding capacity. Previous research investigated chicken breast marinated with black carrot juice reported significant increment in water holding capacity and decline in cooking loss i.e., 24.8 and 17% in contrast to control i.e., 22.2 and 25.9%. They further depicted increment in springiness i.e., 0.77 mm and decline in chewiness i.e., 56.9 N versus control i.e., 0.66 mm and 58.6 N (Çelik et al., 2025). Another study Unal et al. (2023) reported similar results for cow meat marinated with grapes vinegar expressed notable reduction in hardness, chewiness, cohesiveness values i.e., 155.9 N, 52.5 N, 0.52 and increment in springiness value i.e., 0.65 when compared with control i.e., 204.7 N, 64.4 N, 0.54 and 0.62, respectively. Sulaiman et al. (2025) presented chicken breast marinated with 3% lime juice expressed significant higher water holding capacity and cooking loss i.e., 45 and 32.5% against control i.e., 44.4 and 27%. The decline in hardness, chewiness and gumminess values of beef samples marinated with *Syzygium cumini* concentrate may be connected with structural changes of muscle matrix caused by marination. The bioactive compounds (i.e., phenolics, flavonoids) present in *Syzygium cumini* concentrate and acidic environment caused by organic acids of *Syzygium cumini* may alter the muscles protein and muscle fibrillar structures (Serdaroglu et al., 2024). Moreover, the scanning electron microscopy analysis was also investigated in this study (Fig. 5), which revealed notable alterations in the arrangements of muscle fibers. This alteration may be connected with lower hardness and enhanced textural attributes such as improved tenderization of marinated beef samples. However, the advance characterization of protein structure and protein electrophoresis-based analysis needs to investigated for confirming protein conformation or denaturation changes in samples (Nawaz et al., 2024). The increase in cohesiveness and springiness may also be attributed to controlled proteins unfolding's and mild denaturation which may expose reactive groups and support protein-protein interaction, results in more elastic and cohesive network (Liu et al., 2025). Moreover, marinated samples showed reduction in cooking loss could be directly linked with enhanced water retention within the muscle matrix. Marination improves water-protein bindings and decrease moisture expulsion during heating as discussed by (Li et al., 2024). The higher water holding capacity may be accompanied by ionic strength changes, pH modification and protein structural alterations. Marination may shift the pH of muscles far from its isoelectric points, incrementing net charge and supporting water binding sites on protein, leading towards improved water holding capacity (Cornet et al., 2021).

**Fig. 1:**
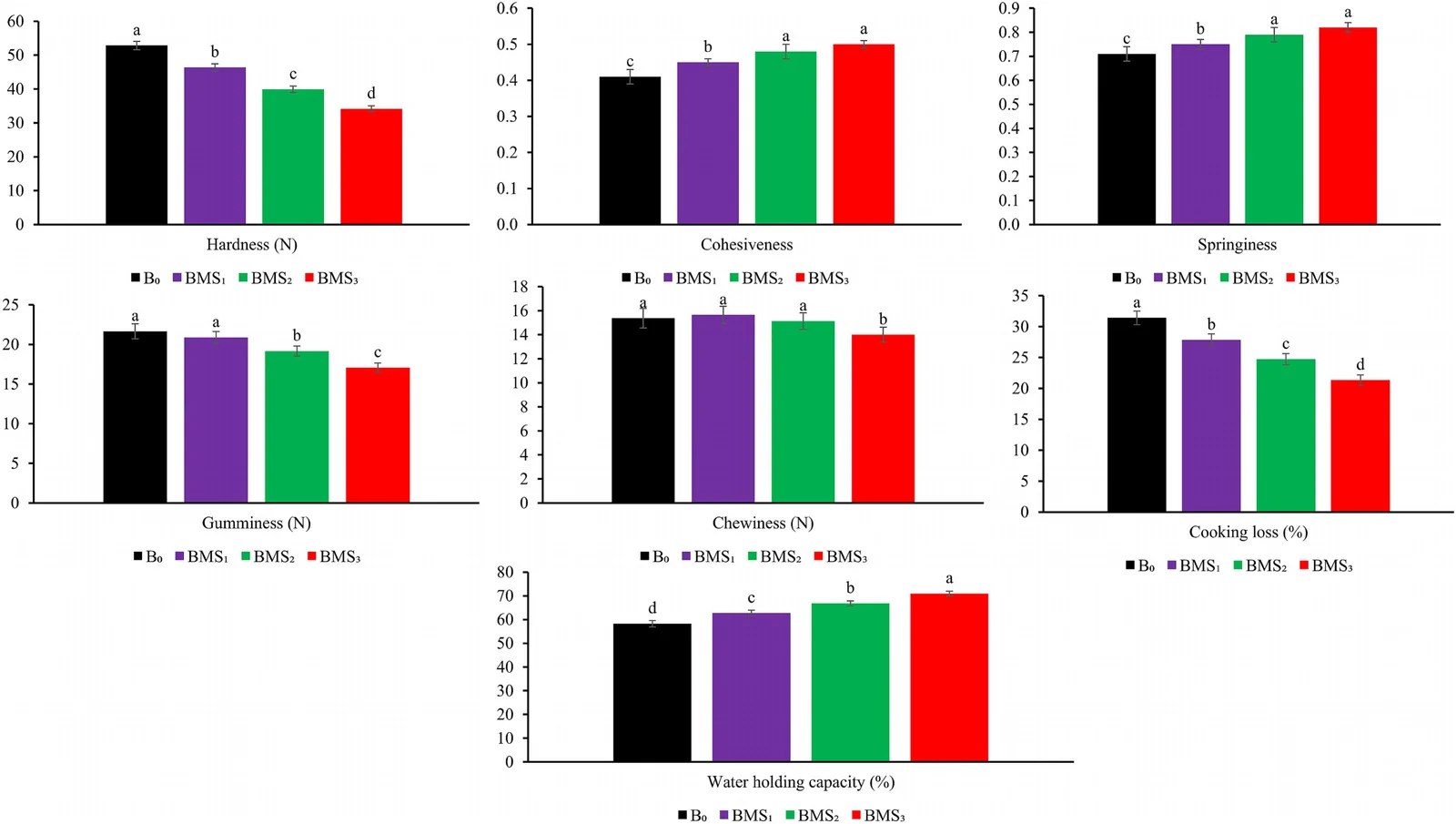
Effect of *Syzygium cumini* concentrate marination on the texture profile analysis, cooking loss and water holding capacity of beef samples. B_0_ = Beef without marination. BMS_1_ = Beef marinated with *Syzygium cumini* concentrate for 1 h, BMS_2_ = Beef marinated with *Syzygium cumini* concentrate for 2 h, BMS_3_ = Beef marinated with *Syzygium cumini* concentrate for 3 h.

### 3.4 Effect of *Syzygium cumini* concentrate marination on pH of beef samples

The pH results reported significant controlled increment (*p* *<* *0.05*), i.e., 5.6–5.5 for BMS_1_-BMS_3_ at 0–9 days of storage, respectively. However, control (B_0_) showed a significantly greater increase (*p* *<* *0.05*) in pH values from 5.8 to 5.9 as contrasted to other all treated samples, over 0 to 9 days of storage (Fig. 2). The beef sample BMS_3_ delineated significant lowest pH values i.e., 5.4–5.5, while control B_0_ elucidated highest pH values i.e., 5.8–5.9 as contrasted to other samples at 0–9 days, sequentially. The beef sample BMS_3_ reported best pH results i.e., 5.4–5.5 against other all samples placed for storage period of 0–9 days. Conclusively, results revealed that increment in *Syzygium cumini* concentrate marination time i.e., 1–3 h notably reduced the pH values of beef samples all over the storage days. Compared to previous studies, Javan et al. (2025) investigated beef steaks marinated with 25 mL soy sauce showed similar lower pH values i.e., 5.1–5.3 against control sample which presented higher values i.e., 5.2–5.8 at ageing time of 1–48 h. Another research experimented on chicken breast marinated with plum juice, red beet juice and black carrot juice presented lower pH values i.e., 4.2, 5.6 and 5.5 in relative to control sample which showed higher pH i.e., 6 as tabulated by (Çelik et al., 2025). Another research also reported similar findings for poultry meat marinated with apple juice, apple + lime juice and lime juice depicted lower pH values i.e., 5.9, 5.8 and 5.9 as assed by control which showed higher value i.e., 6.1 (Augustyńska-Prejsnar et al., 2023). The notable pH increment at storage among beef samples could be connected to aggregation of alkaline nitrogenous compounds i.e., ammonia and amines because of microbial attack and enzymatic reactions as presented by (Javed et al., 2025). Moreover, the lower pH values estimated in marinated beef samples is primarily because of diffusion of phenolic compounds and organic acids from *Syzygium cumini* concentrate into beef muscle tissues. This phenomena contributes in increasing the concentration of hydrogen ions which directly leads to lower the pH values of beef samples (Çelik et al., 2025). The beef sample marinated for 3 h elucidated lowest pH value because longer marination time increased the incorporation of acid in samples, thereby resulting in lower pH values. Additionally, plant-based marinades exhibit antimicrobial activity and retards microbial spoilage and stabilizing the pH by limiting the alkaline compounds formation. In comparison, higher pH estimated in control sample could be accompanied with the microbial proteolysis and deposition of metabolites such as ammonia and ammines discussed in (Javed et al., 2025; Latoch et al., 2023).

**Fig. 2:**
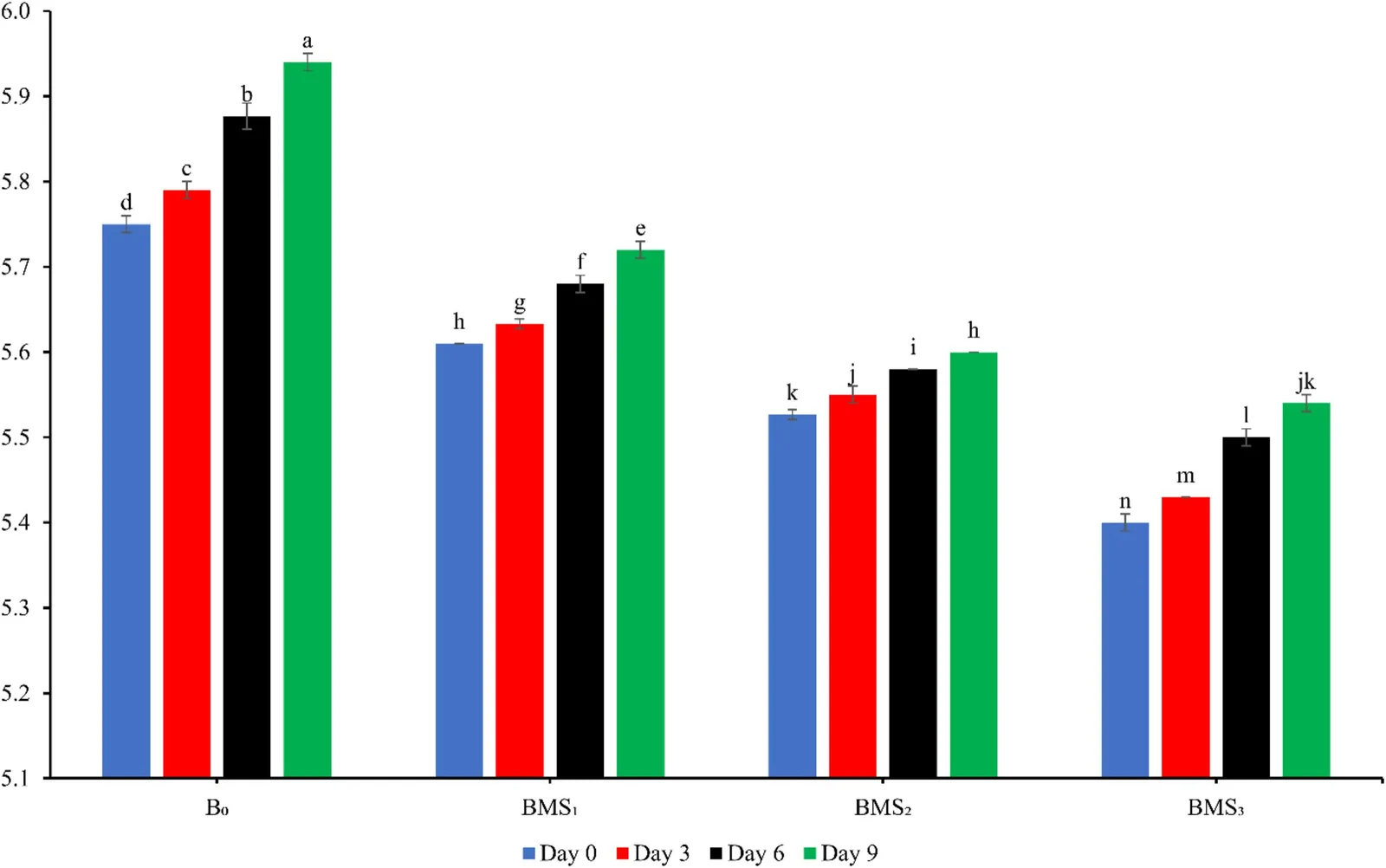
Effect of *Syzygium cumini* concentrate marination on the pH values of beef at 0–9 days of storage. B_0_ = Beef without marination. BMS_1_ = Beef marinated with *Syzygium cumini* concentrate for 1 h, BMS_2_ = Beef marinated with *Syzygium cumini* concentrate for 2 h, BMS_3_ = Beef marinated with *Syzygium cumini* concentrate for 3 h.

### 3.5 Effect of *Syzygium cumini* concentrate marination on TBARS of beef

The TBARS results elucidated significant controlled increase (*p* *<* *0.05*) in values for BMS_1_ to BMS_3_ from 0.9 to 1.4 mg MDA/kg at storage days of 0th to 9th, correspondingly (Fig. 3). Conversely, control showed significant higher increment (*p* *<* *0.05*) in TBARS values i.e., 1.2–2.6 at storage days of 0–9, sequentially. The marinated beef BMS_3_ depicted lowest TBARS value i.e., 1.4 mg MDA/kg, while control showed highest TBARS value i.e., 2.6 mg MDA/kg in comparison to all samples at 9th day of storage. The beef sample marinated with *Syzygium cumini* concentrate for 3 h (BMS_3_) depicted best results for TBARS estimation i.e., 0.6–1.4 mg MDA/kg at 0–9 storage days, respectively. The results dominantly revealed that increase in marination time significantly contributed for reducing the TBARS values of beef samples. Javan et al. (2025) determined the marination effect of 15 mL broccoli juice +25 mL soy sauce on beef, which depicted lower TBARS values i.e., 0.05–0.06 against the control sample i.e., 0.07–0.09 mg MDA/kg for 1–48 h ageing time. Another research reported beef samples marinated with 50 mL unripe grape juice +0.1 g thyme-based marinade solution depicted lower TBARS values i.e., 0.22–0.13 versus the control sample presented i.e., 0.13–0.20 for time interval 2–48 h (Turp et al., 2025). Additionally, one more research study reported chicken breast samples marinated with pomegranate, red beet and black carrot juice expressed lower TBARS readings i.e., 1.6, 1.4 and 1 mg MDA/kg, alongside control presented higher value i.e., 1.8 mg MDA/kg (Çelik et al., 2025). Meat contains poly unsaturated fatty acids and vulnerable to lipid oxidation; considered one of major reason for discoloration, rancid odor, and abnormal flavors in meat. Oxidative reactions generates toxic compounds (cholesterol oxidation products, malondialdehyde, free radicals) that can influence consumers health (Djenane et al., 2023). Results depicted a continues increase in TBARS over the storage intervals could be attributed to existence of fatty acids in higher concentration which are considered to be slightly more vulnerable to lipid oxidation as revealed by (Suleman, Choudhary, Garcia-Galicia, et al., 2025). The lower TBARS values recorded in beef marinated with *Syzygium cumini* concentrate could be accompanied by the effectiveness of bioactive compounds i.e., anthocyanins (delphinidin, cyanidin, petunidin), flavonoids (quercetin, kaempferol) and phenolic acids (gallic acid, ellagic acid) (do Nascimento-Silva et al., 2022). These bioactive compounds found in *Syzygium cumini* concentrate may contribute its role in retardation of lipid oxidation by scavenging free radicals (Nour, 2022). Additionally, some variations in TBARS values from literature may also be linked with distinct meat varieties, varied storage and processing conditions, climate characteristics and makeup for genetic structure (Suleman, Choudhary, Garcia-Galicia, et al., 2025). The lower TBARS results in marinated samples may also explains the improvements in color and sensory attributes of beef at storage. Discoloration, rancid odor and undesirable flavor may be connected to the production of secondary oxidation products during oxidation at storage. However, reduced oxidation can limit the production of such secondary products and prevents the samples from adopting undesirable attributes; thereby results in contributing better preservation of sensory and visual quality (Turp et al., 2025).

**Fig. 3:**
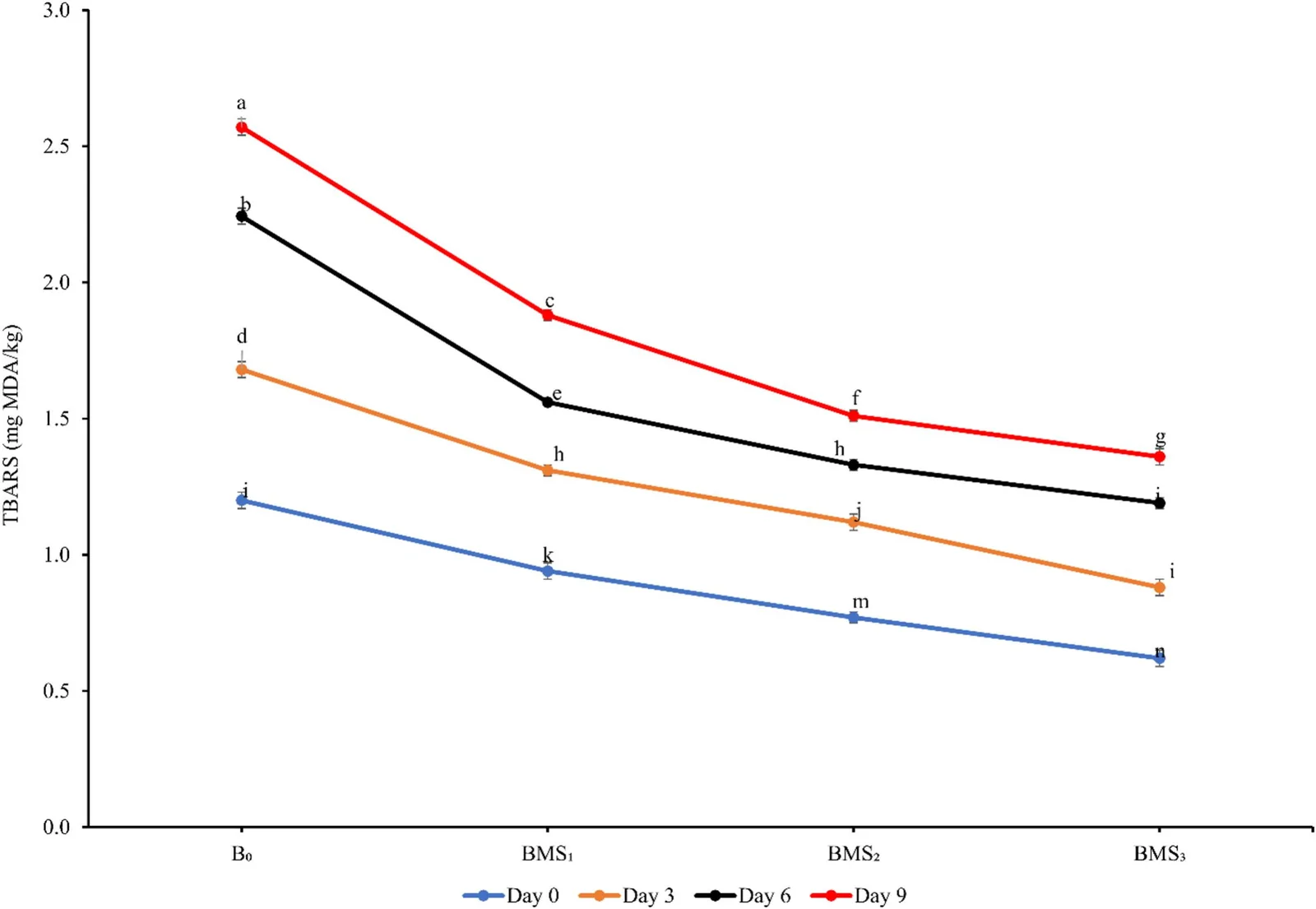
Effect of *Syzygium cumini* concentrate marination on the TBARS values of beef at 0–9 days of storage. B_0_ = Beef without marination. BMS_1_ = Beef marinated with *Syzygium cumini* concentrate for 1 h, BMS_2_ = Beef marinated with *Syzygium cumini* concentrate for 2 h, BMS_3_ = Beef marinated with *Syzygium cumini* concentrate for 3 h.

### 3.6 Effect of *Syzygium cumini* concentrate marination on microbial stability of beef

The microbial results elucidated restrained increment (*p* *<* *0.05*) in total plate counts, *E. coli* and *S. aureus* counts i.e., 3.9–4.4, 1.4–1.5 and 1.7–2.1 Log_10_ CFU/g for BMS_1_ to BMS_3_, at storage days of 0–9, respectively (Table 3). As opposed, control showed significant higher increase (*p* *<* *0.05*) in total plate counts, *Escherichia coli* (*E. coli*) and *Staphylococcus aureus* (*S. aureus*) counts i.e., 4–5.4, 1.6–2.3 and 1.9–3.3 Log_10_ CFU/g at storage days of 0–9, within the same order. BMS_3_ exhibited significant lowest total plate counts, *E. coli* and *S. aurefus* counts i.e., 4.4, 1.5 and 2.1 Log_10_ CFU/g, while control depicted highest counts i.e., 5.4, 2.3 and 3.3 Log_10_ CFU/g, when stored at 9th day of storage. Moreover, BMS_3_ delineated best results for total plate counts, *E. coli* and *S. aureus* counts i.e., 3.7–4.4, 1–1.52 and 1.2–2.1 Log_10_ CFU/g at storage of 0–9 days, consecutively. These findings revealed that *Syzygium cumini* marination improved microbiological stability by retarding microbial proliferation at refrigerated storage. The previous studies reported similar outcomes, wherein, Djenane et al. (2023) reported rabbit meat marinated with Algerian-type spices mixture-based marinade depicted notable decline in total plate counts i.e., 3.5–3.1 in relative to the control sample prepared without marination showed i.e., 3.5–6.4 Log_10_ CFU/g at storage of 0–10 days. Yerli̇kaya et al. (2026) experimented on beef meat marinated with prepared marinade solution-based on strawberry fruit presented no detectable growth of total mesophilic aerobic bacteria and total psychrophilic bacteria i.e., not detected (N.D), while control sample showed counts i.e., 5.5 and 3 Log_10_ CFU/g. Similarly, in another experimental study researchers investigated turkey meat marinated with butter milk and acid whey demonstrated lower mesophilic microbial counts i.e., 2.7 and 3 Log_10_ CFU/g with respect to control i.e., 4.3 Log_10_ CFU/g (Augustyńska-Prejsnar et al., 2023). The estimated notable lower microbial counts in beef marinated samples may be connected with various microbiological and biochemical mechanisms. Firstly, *Syzygium cumini* is considered to enrich with phenolic compounds i.e., anthocyanins, tannins and flavonoids which contributes prominent antimicrobial activity. The lower counts of presumptive *Escherichia coli* and *Staphylococcus aureus* observed in marinated beef may be associated with the antimicrobial potential of phenolic compounds, anthocyanins, tannins, and flavonoids present in *Syzygium cumini* concentrate. However, the present enumeration-based approach does not establish the specific mechanisms responsible for these differences and does not confirm the elimination of pathogenic bacteria (Pillanjinayya et al., 2025; Yerli̇kaya et al., 2026). Secondly, *Syzygium cumini* marinade has acidic nature which lowers the pH of marinated beef samples and creates unfavorable circumstances for the growth of microbes and retards microbial proliferation. Most pathogenic and spoilage causing bacteria prefer pH near to neutral for favorable growth, therefore even slight acidifications may diminish their growth (Kaur et al., 2024). Moreover, beef sample marinated for 3 h revealed lowest microbial counts, because marination for longer time allows more effective penetration of bioactive compounds into meat muscle tissues (Hewage et al., 2022). Further, longer marination improved diffusion and enhanced the contact between antimicrobial agents and microbes which was embedded in meat matrix (Augustyńska-Prejsnar et al., 2023; Djenane et al., 2023). The results indicate that *Syzygium cumini* concentrate marination was associated with lower microbial counts during refrigerated storage, suggesting improved microbial stability under the experimental conditions. However, these findings do not establish the confirmed microbiological safety of the treated beef and does not demonstrate a reduction in foodborne illness risk. As this research was not a pathogen-targeted experimental study and detection of microbial counts was primarily based on the counts from selective media (Javeria et al., 2026). The microbial outcomes were found in relationships with the changes in pH values, especially BMS_3_ which revealed lower microbial counts, with the decrease in pH values. Synergistic influence of antimicrobial compounds and acidic conditions may have reduced proliferation of microbes at storage. Reduction in microbial proliferation may also be connected to enhanced sensory acceptability for marinated beef samples by limiting texture deterioration, off-odor and undesirable metabolites (Ngongoni et al., 2025).

**Table 3:** Effect of *Syzygium cumini* concentrate marination on the total plate counts, *E. coli* counts and *S. aureus* counts (Log_10_ CFU/mL) of beef at 0–9 days of storage.

		Days		
	0th	3rd	6th	9th
Total plate counts				
B_0_	4.07 ± 0.02^l^	4.51 ± 0.03^g^	5.10 ± 0.01^b^	5.44 ± 0.04^a^
BMS_1_	3.92 ± 0.01^m^	4.33 ± 0.01^i^	4.72 ± 0.02^d^	4.92 ± 0.01^c^
BMS_2_	3.81 ± 0.03^n^	4.21 ± 0.02^k^	4.56 ± 0.01^f^	4.62 ± 0.03^e^
BMS_3_	3.70 ± 0.04^o^	3.90 ± 0.03^m^	4.26 ± 0.01^j^	4.40 ± 0.02^h^
*E. coli* counts				
B_0_	1.63 ± 0.06^def^	1.80 ± 0.04^bcd^	1.97 ± 0.03^b^	2.27 ± 0.04^a^
BMS_1_	1.36 ± 0.10^hij^	1.56 ± 0.07^efg^	1.73 ± 0.05^cde^	1.86 ± 0.03^bc^
BMS_2_	1.20 ± 0.17^j^	1.40 ± 0.10^ghi^	1.55 ± 0.13^efgh^	1.73 ± 0.05^cde^
BMS_3_	1.00 ± 0.00^k^	1.26 ± 0.24^ij^	1.32 ± 028^ij^	1.52 ± 0.07^fgh^
*S. aureus* counts				
B_0_	1.92 ± 0.03^g^	2.30 ± 0.02^e^	2.89 ± 0.02^b^	3.25 ± 0.02^a^
BMS_1_	1.73 ± 0.05^h^	2.08 ± 0.04^f^	2.48 ± 0.03^d^	2.73 ± 0.05^c^
BMS_2_	1.52 ± 0.07^i^	1.77 ± 0.07^h^	2.17 ± 0.02^f^	2.41 ± 0.02^d^
BMS_3_	1.20 ± 0.17^k^	1.36 ± 0.10^j^	1.80 ± 0.04^h^	2.12 ± 0.05^f^

Note: Results are depicted as mean ± standard deviations (SD). The superscript lettering on the values demonstrated significant results among the means on storage in rows and treatments in column. B_0_ = Beef without marination. BMS_1_ = Beef marinated with *Syzygium cumini* concentrate for 1 h, BMS_2_ = Beef marinated with *Syzygium cumini* concentrate for 2 h, BMS_3_ = Beef marinated with *Syzygium cumini* concentrate for 3 h.

### 3.7 Effect of *Syzygium cumini* concentrate marination on sensorial properties of beef

Sensory properties of cooked marinated beef exhibited significant reduction (*p* *<* *0.05*) in sensory score of appearance, aroma, tenderness, juiciness, flavor and overall acceptability i.e., 8–6.3, 8.2–5.6, 8–6, 7.3–5.3, 8–5.3 and 7.9–5.7 for BMS_1_ to BMS_3_ at 0–9 storage days, respectively (Table 4). However, control depicted significant higher decline (*p* *<* *0.05*) in sensory scores for appearance, aroma, tenderness, juiciness, flavor and overall acceptability i.e., 7.7–5.8, 7–4.7, 7.3–4.7, 7–4.7, 7–4.2 and 7.2–4.8 at 0–9 storage days, sequentially. Moreover, BMS_2_ depicted highest sensory scores for appearance, aroma, tenderness, juiciness, flavor and overall acceptability i.e., 7, 7, 7.3, 6.3, 7 and 6.9, while B_0_ depicted lowest scores i.e., 5.8, 4.7, 4.7, 4.7, 4.2 and 4.8 at 9th day of storage. In addition, BMS_2_ showed best sensory scores for appearance, aroma, tenderness, juiciness, flavor and overall acceptability i.e., 8.3–7, 8.7–7, 8.7–7.3, 8–6.3, 8.7–7 and 8.5–6.9 when stored at 0–9 days of storage, within the same order. The increment in marination time overall increased the sensory scores of cooked marinated beef samples as compared to control. Earlier studies reported that Nour (2022) investigated pork meat marinated with plum juice elucidated prominent highest sensory scores for color, tenderness, juiciness, taste, flavor and overall acceptability i.e., 7.7–6.4, 7.3–6.3, 6.8–5.3, 8.7–6.6, 8.7–6.9 and 8.4–6.8 with respect to control i.e., 8.3–5.7, 7.3–5.9, 6.8–4.9, 7.8–5.4, 7.8–4.8 and 7.6–4.5 at storage of 0–12 days, respectively. In another research, chicken breast was marinated with the pomegranate juice represented higher sensory scores for color, texture, flavor and overall acceptability i.e., 7, 8, 6, 7 as relative to control sample i.e., 8, 6, 5, 6, when investigated on 9-point hedonic scale (Çelik et al., 2025). Another research explored the strawberry-based marinade marination influence on the beef samples, results stated significant higher sensory readings for color, texture, odor, flavor and overall acceptability i.e., 7.8, 7.4, 8, 7.6 and 7.8 as opposed to the control i.e., 5.8, 6.8, 7.8, 7.2 and 7.6 (Yerli̇kaya et al., 2026). Scientifically, the reduction in sensory ratings estimated in beef samples over the storage period could be due to biochemical degradation and microbial proliferation. The microbe's growth in meat samples leads to development of slimy texture, off-odor and undesirable metabolites which influence negatively on flavor, aroma and overall acceptability (Augustyńska-Prejsnar et al., 2023; Djenane et al., 2023). Moreover, oxidation reactions can produce aldehyde, ketone and other secondary products which are connected to discoloration and rancid flavor, while proteolytic changes reduce juiciness, tenderness and weaken the muscles structure (Wang et al., 2021). As contrasted, higher sensory readings were estimated in marinated beef samples could be linked with the existence of natural antimicrobial and antioxidants compounds i.e., anthocyanins and phenolics in *Syzygium cumini* (Qamar et al., 2022). Additionally, the acidic elements of *Syzygium cumini* marinade supports swelling of proteins, retention of moisture and enhancing palatability (Sristi et al., 2025). Further, this marination also improves the water holding capacity of beef samples, results in induction of structural modifications in protein muscle, leading towards improved juiciness and tenderness (Ji et al., 2024).

**Table 4:** Effect of *Syzygium cumini* concentrate marination on the sensory profiling of beef at 0–9 days of storage.

		Days		
	0th	3rd	6th	9th
Appearance				
B_0_	7.67 ± 0.99^abcd^	7.00 ± 1.00^cde^	6.40 ± 0.09^ef^	5.80 ± 0.34^f^
BMS_1_	8.00 ± 1.00^abc^	7.89 ± 0.22^abcd^	7.33 ± 0.58^bcde^	6.70 ± 1.00^cde^
BMS_2_	8.33 ± 0.58^ab^	8.00 ± 1.00^abc^	7.67 ± 1.00^abcd^	7.00 ± 0.00^def^
BMS_3_	8.67 ± 0.58^a^	7.43 ± 0.57^bcde^	7.11 ± 0.84^cde^	6.29 ± 0.17^ef^
Aroma				
B_0_	7.00 ± 0.00^cd^	6.15 ± 0.79^de^	5.33 ± 0.58^ef^	4.67 ± 0.58^f^
BMS_1_	8.23 ± 0.83^ab^	7.89 ± 0.22^abc^	7.00 ± 1.00^cd^	6.30 ± 0.53^de^
BMS_2_	8.67 ± 0.58^a^	8.39 ± 0.92^ab^	7.66 ± 1.03^abc^	7.00 ± 1.00^cd^
BMS_3_	7.67 ± 0.58^abc^	7.19 ± 0.16^bcd^	6.44 ± 0.51^de^	5.57 ± 1.13^ef^
Tenderness				
B_0_	7.33 ± 1.53^abcde^	7.00 ± 0.00^bcde^	6.33 ± 0.58^de^	4.67 ± 0.58^f^
BMS_1_	8.00 ± 1.00^abc^	7.73 ± 0.90^abcd^	7.00 ± 0.00^bcde^	6.67 ± 1.53^cde^
BMS_2_	8.67 ± 0.58^a^	8.23 ± 1.04^ab^	8.00 ± 1.00^abc^	7.33 ± 1.53^abcde^
BMS_3_	7.67 ± 0.58^abcd^	8.00 ± 0.00^abc^	6.60 ± 0.10^cde^	6.00 ± 0.00^ef^
Juiciness				
B_0_	7.00 ± 1.00^abcd^	6.33 ± 0.13^bcdef^	5.00 ± 1.00^fg^	4.73 ± 0.21^g^
BMS_1_	7.33 ± 0.58^abc^	7.00 ± 0.00^abcd^	6.33 ± 1.53^bcdef^	5.75 ± 1.00d^efg^
BMS_2_	8.00 ± 0.00^a^	7.33 ± 0.58^abc^	7.00 ± 0.00^abcd^	6.33 ± 1.53^bcdef^
BMS_3_	7.67 ± 1.15^ab^	6.67 ± 1.15^abcde^	6.00 ± 1.00^cdefg^	5.33 ± 1.15^efg^
Flavor				
B_0_	7.00 ± 1.00^bcd^	6.33 ± 0.58^cde^	5.11 ± 0.85^fg^	4.17 ± 0.29^g^
BMS_1_	8.00 ± 1.00^ab^	7.43 ± 0.98^bc^	7.00 ± 0.00^bcd^	6.00 ± 1.00^def^
BMS_2_	8.67 ± 0.58^a^	8.00 ± 0.00^ab^	7.67 ± 0.58^ab^	7.00 ± 0.00^bcd^
BMS_3_	7.56 ± 0.66^ab^	6.00 ± 1.00^def^	5.78 ± 0.70^ef^	5.33 ± 0.21^ef^
Overall acceptability				
B_0_	7.20 ± 0.12^cd^	6.56 ± 0.35^def^	5.63 ± 0.57^h^	4.81 ± 0.18^i^
BMS_1_	7.91 ± 0.39^ab^	7.59 ± 0.09^bc^	6.93 ± 0.31^de^	6.28 ± 0.47^fg^
BMS_2_	8.47 ± 0.42^a^	7.99 ± 0.29^ab^	7.60 ± 0.58^bc^	6.93 ± 0.70^de^
BMS_3_	7.85 ± 0.33^ab^	7.06 ± 0.13^cd^	6.39 ± 0.25^ef^	5.71 ± 0.35^gh^

Note: Results are depicted as mean ± standard deviations (SD). The superscript lettering on the values demonstrated significant results among the means on storage in rows and treatments in column. B_0_ = Beef without marination. BMS_1_ = Beef marinated with *Syzygium cumini* concentrate for 1 h, BMS_2_ = Beef marinated with *Syzygium cumini* concentrate for 2 h, BMS_3_ = Beef marinated with *Syzygium cumini* concentrate for 3 h.

### 3.8 Principal component analysis (PCA)

The first two principal components i.e., PC1 and PC2 expressed total variance of 79.03% including PC1 (65.77%) and PC2 (13.26%) Fig. 4 (a). PC1 variedly separates quality parameters indicating that positive side of PC1 is prominently connected with total plate counts, *E. coli*, *S. aureus*, TBARS and pH. These parameters are grouped closely and oriented in the same direction which indicates significant positive association among these parameters. These interconnections may be attributed to the associated nature of biochemical degradation and proliferation of microbes in beef. The increase in microbes can promote metabolic and proteolytic reactions that can produce nitrogenous metabolites of alkaline nature which results in elevation of pH. Similarly, endogenous enzyme-based and microbial activities may support oxidative reactions containing membrane lipids, which contributes to enhanced lipid peroxidation and TBARS value. Hence, microbial proliferation, TBARS and pH represents the corresponding progression of oxidative and microbial deterioration over storage (Javeria et al., 2026; Suleman et al., 2023). As contrasted, sensory characteristics are positioned on the PC1 negative side along with redness (*a*^*⁎*^) depicting dominant inverse relationship with spoilage indicators. PC1 can also be explained as a quality deterioration axis where directional movement towards positive side expressing progressive spoilage, and negative side depicted superior freshness and sensory quality. The reduction in sensory quality over storage may be linked with pigment alterations, microbial metabolites accumulation and lipid oxidation products which jointly contributes to unwanted texture, discoloration, odor and flavor. Therefore, the *Syzygium cumini* treated samples movements towards the PC1 negative side may be associated with antimicrobial and antioxidant activities because of its anthocyanins, flavonoids and phenolic compounds. Mentioned compounds may also scavenge free oxygen radicals and suppress microbial growth which slow down the biochemical reactions linked with deterioration of quality (Qamar et al., 2022). The second principal component (PC2) mainly captures variations linked with color attributes i.e., yellowness (*b*^*⁎*^) depicting strong positive loading along the vertical axis, while the lightness (*L*^*⁎*^) and pH contributes in the negative direction moderately, also discussed in (Yerli̇kaya et al., 2026). This represents that PC2 differentiates samples based on changes estimated in color, especially the yellowness development during storage. Additionally, spatial partition of treatments further confirms these associations. Control sample are predominantly positioned on the positive side of PC1 which are strongly associated with the oxidative and microbial properties estimating higher quality deterioration. As opposed, *Syzygium cumini* treated samples (BMS_1_, BMS_2_, BMS_3_) presented shifting towards negative-side of PC1 which depicts observable connections with sensory attributes; thereby elucidating enhanced quality preservation (Zheng et al., 2024). This separation could be associated with combined influence of *Syzygium cumini* concentrate bioactive compounds and acidic environment which may retard proliferation of microbes and oxidative stress at storage. Among all treatments, BMS_2_ elucidated strong correlation with sensorial and chromatic attributes suggesting higher effectiveness in ensuring product quality. Overall, PCA outcomes revealed the coordinated relationships among the sensory deterioration, lipid oxidation, physicochemical changes and proliferation of microbes (Javeria et al., 2026). While the favorable positioning of sample marinated with *Syzygium cumini* comparatively delayed these associated deterioration process. Furthermore, the storage time progression is evident from partition of sample points where day 0 samples cluster existed close to sensory and freshness-linked region. However, samples from last storage days showed clear shift towards region recognized by higher lipid oxidation and microbial load. This trend revealed that increase in storage time prominently increased deterioration process, but marinated samples delineates comparatively slower shift towards spoilage region as opposed to control (Huerta-Jimenez et al., 2025). Overall, the PCA results illustrated associations among microbial counts, lipid oxidation, physicochemical characteristics, and sensory attributes, with the observed patterns suggesting comparatively slower quality deterioration in marinated samples during refrigerated storage.

**Fig. 4:**
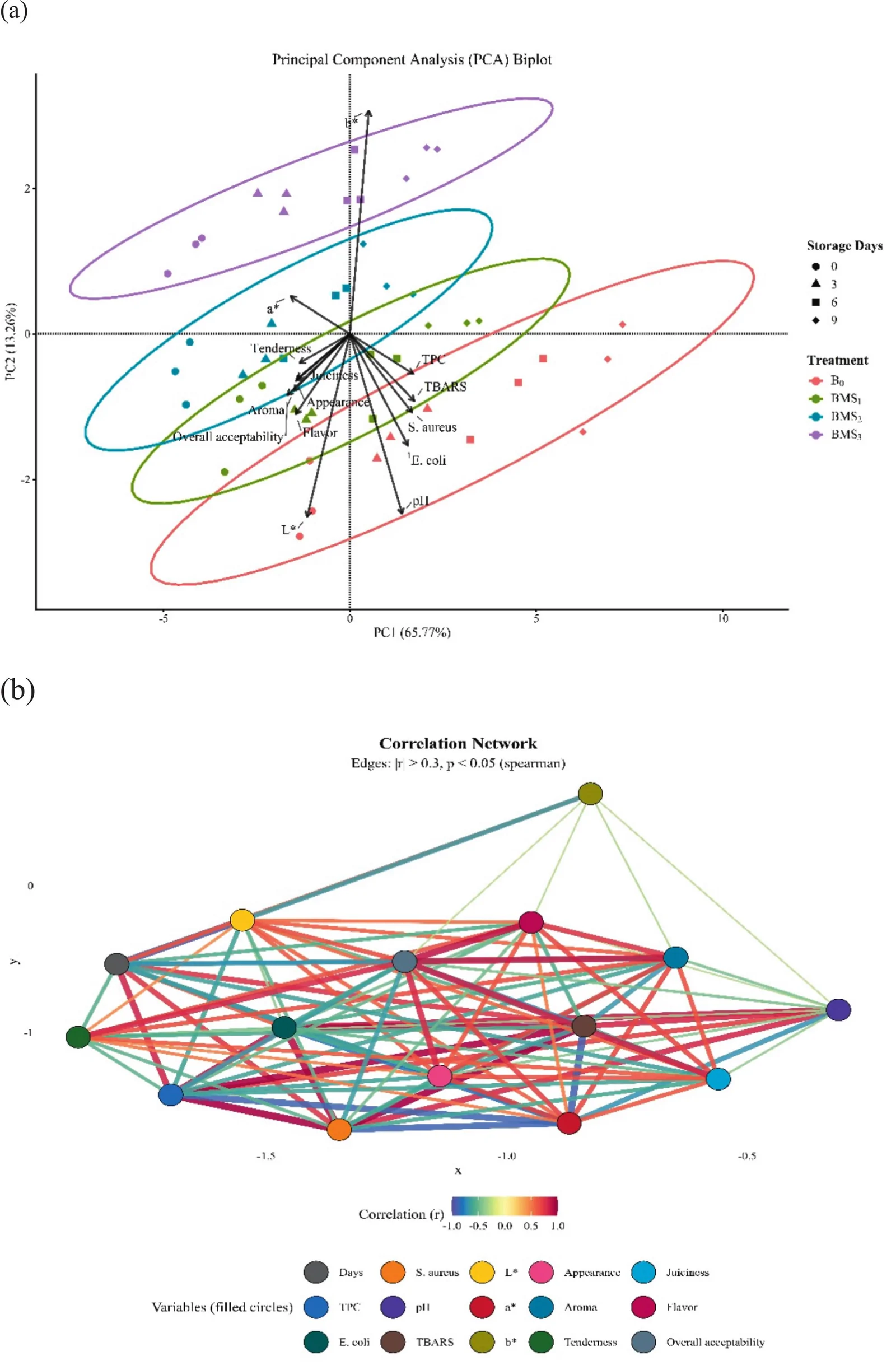
(a) Principal component analysis (b) Correlation analysis of beef samples marinated with *Syzygium cumini* concentrate. TPC: Total plate counts, TBARS: Thiobarbituric Acid Reactive Substances, L^⁎^: Lightness, a^⁎^: redness, b^⁎^: yellowness. B_0_ = Beef without marination. BMS_1_ = Beef marinated with *Syzygium cumini* concentrate for 1 h, BMS_2_ = Beef marinated with *Syzygium cumini* concentrate for 2 h, BMS_3_ = Beef marinated with *Syzygium cumini* concentrate for 3 h.

### 3.9 Correlation analysis

The correlation network analysis depends on the spearman's rank correlation i.e., (|r| ≥ 0.3, *p* < 0.05) delineates a brief overview of inter-relationships among all parameters. In this network plot, nodes indicates variables, while edges represent significant correlation, edge thickness revealing the association strength and color denoting the directions (positive or negative) Fig. 4 (b). The network plot elucidated that total plate counts, *E. coli* and *S. aureus* had substantial positive correlation among each other and also with pH and TBARS results. Positive relationships among these variables represents the concurrent oxidative and microbial deterioration. Microbial growth can enhance proteolytic and metabolic actions in meat, which cause alkaline metabolites production and pH consequent increase. This increase in pH supports several spoilage microorganism growths; thereby developing a deteriorative cycle. Further, endogenous enzymatic and microbial activities may attribute to oxidation products formation i.e., malondialdehyde, which results in increased TBARS values (Saeed et al., 2026). Notably, these sensory characteristics depicted prominently negative correlation with the pH, TBARS, and microbial spoilage, as represented by contrasting edge color. The negative relationships estimated between sensory characteristics and oxidative and microbial indicators may be discussed by the lipid oxidation products and soilage metabolites accumulation. These compounds can generate undesirable flavor and odor, and further contributes in deterioration and discoloration of textural attributes; thereby decreasing sensory acceptability (Shahidi & Hossain, 2022; Yi et al., 2025). As comparative to this, higher sensory ratings of *Syzygium cumini* treated samples could be linked with the antimicrobial and antioxidant potential of its flavonoids and phenolic constituents. The underlying mechanism may be attributed to the actions of antimicrobial phytochemicals of *Syzygium cumini* that may interfere with cellular metabolism and cell membranes of microbes; thereby reducing microbial load (Qamar et al., 2022). Additionally, phenolic compounds may interrupt oxidative chain reactions by donating electron or hydrogen atom to reactive species (Siebeneichler et al., 2026). Therefore, this correlation patterns revealed statistical evidence is consistent with the proposed mechanism, that *Syzygium cumini* may provide favorable retarding effects for oxidative deterioration and microbial proliferation. Redness (*a*^*⁎*^) depicted positive correlation with sensory characteristics, indicating its significant role in acceptability of consumers. Lightness (*L*^*⁎*^) delineated moderate positive linkage with the sensory traits, representing that brighter meat is generally perceived as more acceptable and fresher. *Syzygium cumini* is enriched with anthocyanins and phenolic constituents which may influence on the oxidative process and pigment stability connected to meat discoloration. Hence, correlation illustrated that beef quality improvements involves the interconnected changes in color, sensory, physicochemical, oxidative and microbial attributes (Javan et al., 2025; Zheng et al., 2024).

### 3.10 Fourier transform infrared (FTIR) spectroscopy

The treatments spectra reported absorption bands connected with proteins, lipids, bioactive compounds and moisture, naturally accompanied in marinating concentrate and beef (Siddiqui et al., 2021). The molecular interactions among the beef and marinating concentrate may be estimated by slight shift in peaks after marination Fig. 5 (a). An intense and broad absorption was estimated in the wavenumber regions i.e., 3269–3365, approximately indicating the O—H stretching of hydroxyl groups and amide compounds-based N—H stretching (Candoğan et al., 2021; Javed et al., 2025). Here, marinated beef represented peaks at wavenumbers 3365.24, 3282.53 and 3327.54 cm^−1^ for BMS_1_, BMS_2_ and BMS_3_, respectively. Conversely, untreated sample B_0_ delineated 3269.90 cm^−1^ wavenumber peak on the FTIR plot (Candoğan et al., 2021). The estimated changes in the broad region of N-H/O-H may be connected to hydrogen bonding modifications involving muscle proteins and OH-containing compounds in *Syzygium cumini* concentrate. Many OH groups are present in phenolic compounds that can participate in non-covalent interactions with protein-based side chains, leading to modifying vibrational behavior and local molecular environment of proteins (Huang et al., 2025; Sharma et al., 2020). Another dominant band absorption was estimated at 1632–1659 cm^−1^, which connected to amide I region primarily linked with C=O vibrational stretching of proteins (Candoğan et al., 2021). The treated sample delineated peaks for BMS_1_ (1652.72 cm^−1^), BMS_2_ (1632.74 cm^−1^) and BMS_3_ (1632.59 cm^−1^) as contrasted to untreated beef (1653.87 cm^−1^) (Huang et al., 2025). The estimated shift in amide-I band indicates probable conformational changes and alterations of protein microenvironment induced by the phenolic rich and acidic exposure of marinade. *Syzygium cumini* organic acids may alter the electrostatic interactions myofibrillar proteins and supports unfolding of protein partially. These structural alterations can weaken the interaction of protein-protein and enhance muscle matrix accessibility to marinade constituents and water. Thereby, giving a plausible molecular explanation for enhanced water retention and tenderness characteristics of marinated beef samples (Huang et al., 2025). Numerous minor bands with distinct intensities were observed at 1500–1000 cm^−1^ approximately among all treatments. Mentioned peaks was generally connected with C—N and C—O stretching and carbohydrate-related vibrations (Candoğan et al., 2021; Keshavarzi et al., 2020). The slight enhanced complexity and bands intensity in samples after marination may be associated with sugars, bioactive and organic acid components of *Syzygium cumini* concentrate in matrix of beef. The existence of bioactive may be attributed to enhanced functional properties and oxidative stability in samples after marination (Latoch et al., 2023). Accumulatively, all discussed spectral changes suggested *Syzygium cumini* marination influence may involves non-covalent interactions, conformational changes, protein modifications in the muscle matrix. These molecular alterations represents mechanistic link among the texture improvements and FTIR observations of marinated beef samples (Ardicli et al., 2024).

**Fig. 5:**
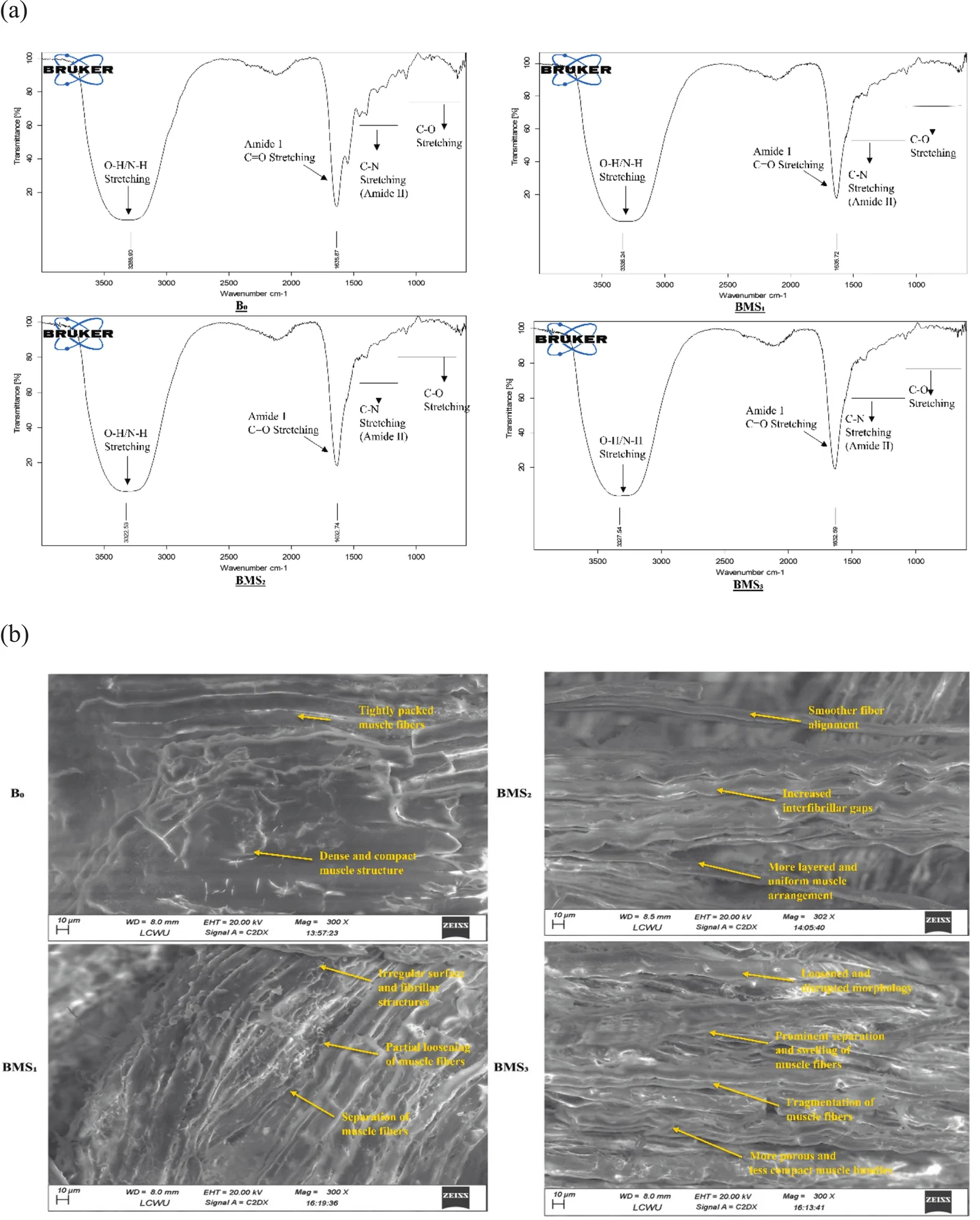
(a) Fourier transform infrared spectroscopy analysis (b) Scanning electron microscope analysis representing compositional properties of beef marinated with *Syzygium cumini* concentrate. B_0_ = Beef without marination. BMS_1_ = Beef marinated with *Syzygium cumini* concentrate for 1 h, BMS_2_ = Beef marinated with *Syzygium cumini* concentrate for 2 h, BMS_3_ = Beef marinated with *Syzygium cumini* concentrate for 3 h.

### 3.11 Scanning electron microscope (SEM) analysis

The influence of marination on the meat fibers were revealed by considerable modification in microstructures among samples Fig. 5 (b). BMS_1_ delineated enhanced irregularities on the surface, fibrillar structures extended, separation and partial loosening of muscle fibers. Compact tissues disruption may be evidenced by insertion of bioactive into muscle matrix by *Syzygium cumini* concentrate. Interactions weakening among connective tissues and partial denaturation of protein may be caused by this insertion. Samples BMS_3_ and BMS_2_ showed more prominent alterations in the meat matrix. BMS_2_ expressed enhancement in smoothness of fiber alignment, enhanced interfibrillar gaps; thereby represented more layered and uniform muscle arrangement than untreated sample. Such microstructural alterations expressed higher diffusion of marinade components into connective meat tissues and increment in muscle integrity breakdown. Likewise, BMS_3_ represented a greater loosened and disrupted morphology with prominent separation, swelling and fragmentation of muscle fibers. More porous, slight compact muscle bundles revealed prominent physicochemical and proteolytic alterations after marination for longer time. The estimated alterations may be connected to phenolic compounds, organic acids and bioactive action of *Syzygium cumini* concentrate (Csurka et al., 2024). These components may interact with muscle protein because of electrostatic bonding and hydrogen interactions which may cause connective tissues and myofibrillar structures weakening (Mazaheri Kalahrodi et al., 2021). Additionally, marinade may create acidic environment which supports unfolding of protein and disruption of muscle matrix, which may increase tissues permeability, contributing in improved tenderness (Lin et al., 2024). The disruption and loosening of muscle fibers with increment in marination may indicate the influence of marinade in improving water retention and overall texture quality of meat (Fan et al., 2024). However, control B_0_ elucidated denser and compact morphology, with irregular agitated regions and tightly packed fibers. Muscle bundles representing the intact muscle tissue structure, limited fibrillar spacing and more rigid structure. These outcomes could be connected to absence of marination in untreated sample. Conclusively, SEM revealed that marination altered beef morphology and structure prominently linked to tenderness, texture and overall quality of product (Ehsanur Rahman et al., 2023).

### 3.12 Limitations and future suggestions

The present research was planned to investigate the impact of *Syzygium cumini* concentrate marination on the storage stability of beef, when marinated for different time intervals in comparison with un-marinated samples. But, this study lacks comparison with pH/acidity-matched marinades and water/base-marinade, to investigate acidity, immersion and specific *Syzygium cumini* bioactive compound influence on beef. Additionally, economic or cost-effectiveness analysis was not investigated, as it was beyond the scope of the present study. Future researches should focus on advance bioactive characterization of marinades, and explore specific compounds influence on the storage quality of meat and meat-based products. Future studies should also evaluate *Syzygium cumini* concentrate-based different concentrations in combination with different marination times to investigate optimized processing conditions. Researches should also be conducted on exploring plant-based marinade effectiveness with the integration of emerging preservation technologies such as nanoencapsulation, and ultrasonication.

## 4 Conclusion

The present study demonstrated that marination of beef with *Syzygium cumini* concentrate influenced its physicochemical, sensory, oxidative, and microbial characteristics during refrigerated storage. Marinated samples exhibited lower TBARS values and microbial counts, along with improvements in several textural properties and sensory attributes compared with the unmarinated control. These findings suggest that *Syzygium cumini* concentrate may contribute to maintaining beef quality and improving microbial stability under the tested laboratory conditions. PCA and correlation analyses further illustrated associations among microbial counts, oxidative changes, physicochemical characteristics, and sensory quality. SEM and FTIR analyses suggested structural and molecular changes associated with marination. Among the tested treatments, BMS_2_ achieved the highest sensory acceptance, whereas BMS_3_ exhibited the most favorable results for several microbial and oxidative indicators. However, the microbiological findings are limited to the enumeration methods employed and do not establish the microbiological safety of the treated beef. Further studies involving validated pathogen-specific methods, additional marinade concentrations, and different processing conditions are needed to clarify the broader preservation potential of *Syzygium cumini* concentrate in meat products.

## CRediT authorship contribution statement

**Muhammad Asad Ali Khan:** Writing – review & editing, Writing – original draft, Visualization, Methodology, Formal analysis, Conceptualization. **Raheel Suleman:** Writing – review & editing, Writing – original draft, Visualization, Supervision, Investigation, Data curation, Conceptualization. **Muhammad Muzamil:** Writing – review & editing, Writing – original draft, Visualization, Software. **Noor-Ul-Huda:** Writing – review & editing, Writing – original draft, Visualization, Software. **Tariq Ismail:** Writing – review & editing, Visualization. **Muhammad Aftab Zahoor:** Writing – review & editing. **Huan Liu:** Writing – review & editing, Visualization. **Moneera O. Aljobair:** Writing – review & editing, Visualization. **Isam A. Mohamed Ahmed:** Writing – review & editing, Visualization.

## Funding

This research was funded by Researchers Supporting Project Number (PNURSP2026R251), Princess Nourah bint Abdulrahman University, Riyadh, Saudi Arabia.

## Declaration of competing interest

The authors declare that they have no competing financial or personal interests that could have appeared to influence the work reported in this paper.

## Data Availability

Data will be made available on request.
